# Evo-devo of human adolescence: beyond disease models of early puberty

**DOI:** 10.1186/1741-7015-11-113

**Published:** 2013-04-29

**Authors:** Ze'ev Hochberg, Jay Belsky

**Affiliations:** 1 Division of Pediatric Endocrinology, Meyer Children's Hospital, Rambam Health Care Campus, Haaliya Street, Haifa 31096, Israel; 2 Rappaport Family Faculty of Medicine, Technion - Israel Institute of Technology, Efron Street, Haifa 31096, Israel; 3 Department of Human and Community Development, University of California Davis, One Shields Avenue, Davis, CA 95616, USA; 4 Department of Special Education, King Abdulaziz University, Rihanat Aljazeera Street, Jeddah, Saudi Arabia; 5 Department of Psychological Science, Birkbeck University of London, Malet Street, London WC1B 3HE, UK

**Keywords:** adolescence, evolution, juvenility, puberty, youth

## Abstract

Despite substantial heritability in pubertal development, much variation remains to be explained, leaving room for the influence of environmental factors to adjust its phenotypic trajectory in the service of fitness goals. Utilizing evolutionary development biology (evo-devo), we examine adolescence as an evolutionary life-history stage in its developmental context. We show that the transition from the preceding stage of juvenility entails adaptive plasticity in response to energy resources, other environmental cues, social needs of adolescence and maturation toward youth and adulthood. Using the evolutionary theory of socialization, we show that familial psychosocial stress fosters a fast life history and reproductive strategy rather than early maturation being just a risk factor for aggression and delinquency. Here we explore implications of an evolutionary-developmental-endocrinological-anthropological framework for theory building, while illuminating new directions for research.

## Introduction

Evolutionary development biology (evo-devo) is concerned with how developmental systems evolved, while probing the consequences of these historically established systems for organismal evolution [1]. Research in evo-devo has formed around comparative embryology and morphology, evolutionary developmental genetics, and experimental epigenetics. Here we examine adolescence from an evo-devo perspective, treating this life-history stage of rapid growth and maturation in its ecological and developmental context [2].

Modern lifestyle and medicine have influenced nutritional and infectious constraints on puberty, resulting in the secular trend in pubertal development over the past 150 years. In girls, more than in boys, the change in pubertal age has been intriguing; in the last decades, the rate of precocious sexual maturation in girls has been high and increasing, and the mechanism for the 'epidemic' has been much debated, pointing fingers at toxins and perhaps other chemical products. Evidence that the timing of boys' somatic maturation is changing has recently been reviewed and remains inconclusive [3], though one Danish study documented a 3-month acceleration in male pubertal onset across a 15-year period (from 11.92 years in 1991 to 11.66 years in 2008) [4].

Evolutionary analysis highlights the fact that it is the female who is reproductively constrained in terms of the maximum number of offspring she can generate over her reproductive years. In consequence, early maturation affords a potential fitness advantage for females more than males, allowing more time to reproduce. Thus, evolutionary life-history thinking challenges the prevailing notion that early puberty is exclusively or primarily pathological in origin, viewing it rather as an adaptive response to changing life conditions. Indeed, as we hope to show, evidence indicates that since the emergence of *homo sapiens *there has been much change in the timing of pubertal maturation - and not just in a singular direction - and a variety of contextual factors appear to regulate pubertal development. To our way of thinking, it is a mistake to focus only on environmental toxins or even simply cast changes in pubertal timing in disease terms.

We challenge the pathological view by advancing an evolutionary perspective on the issue of juvenile transition and timing of pubertal development, as drawn from life-history theory. Toward this end we consider the anthropological record, showing that adolescence as a stage was a new development in primate life history and that over time there have been many changes in pubertal timing, both accelerating and delaying it. While acknowledging heritable individual differences in pubertal timing, we emphasize developmental plasticity and the role of the environment in regulating pubertal timing in the service of fitness goals, using hormonal and developmental mechanisms. A central claim will be that the transition from the preceding stage of juvenility to adolescence entails adaptive developmental response to energy resources, other environmental cues, social needs of adolescence and maturation toward youth and adulthood, with the latter defined as the life-history stage of reproduction (Table 1). The plasticity which we argue characterizes adolescence is regulated by hormonal processes. We explore implications of this evolutionary-developmental-endocrinological-anthropological framework for theory building, while illuminating new directions for research.

**Table 1:** Developmental tasks for adolescents and young adults [67].

The adolescent tasks:	The adult tasks:
(a) integration in the peer group (b) acceptance of physical maturity (c) establishment of an autonomous identity (d) achievement of independence from parents (e) preparation of future family life (f) achievement of sociopolitical awareness (g) preparation for an occupation (h) having a romantic relationship (i) formation of close friendships	(a) taking over responsibility as a citizen (b) the development of a firm partnership (c) living with the partner (d) establishing a family (e) caring for a family (f) starting a career (g) becoming integrated in a social group (h) establishing an independent household

## Conceptual foundations

### Life-history theory

Evolutionary life-history theory deals with the strategic allocation of an organism's energy toward growth, maintenance and reproduction, including raising offspring to independence, while avoiding death [2,5]. It predicts that selection will promote fitness-enhancing physiological, psychological and behavioral mechanisms that make strategic tradeoffs involved in the allocation of energetic resources to influence the three foundations of natural selection: survival, sexual selection and fertility fitness.

Relative to other species, human life-history strategy includes a long period of postnatal growth, including dependency to sexual maturity, rapid adolescent growth and delayed reproduction [5]. Consideration of intermediate growth stages and the transitions between them from a life-history perspective affords insight into strategic objectives that include the age of pubertal onset, pubertal tempo, ultimate size and cognitive targets.

### A matter of definition

The terms puberty and adolescence are often used interchangeably and thus incorrectly. Whereas puberty refers to the activation of the neuroendocrine hypothalamic-pituitary-gonadal axis that culminates in gonadal maturation and the biological effects of sex steroids, the package we call adolescence includes such pubertal development plus the growth spurt, cognitive and brain maturation, and social aspects in learning, intimacy and mutual support, intensification of pre-existing friendships, development of new relationships, and the attainment of biosocial skills needed for successful reproduction. The collective endpoint of the adolescence package is the socially and reproductively mature adult. To promote reproductive and parenting success in the service of reproductive fitness, hormonal and mental maturations are intimately coupled through iterative transactions between the nervous system and endocrine systems, with the latter involving gonadal steroid hormones [6].

### Adolescence as a unique life-history stage

As late as 3,000,000 to 4,000,000 years ago the early homininae *Australopithecus afarensis *had only three postnatal, pre-adult life-history stages, just like the chimpanzee - 5 years of infancy, 5 years of juvenility and 2 years of youth - before the onset of reproduction [2]. During the evolution of the Hominidae, childhood and adolescence were inserted as new life-history stages of *Homo sapiens *[7]: infancy, lasting 30 to 36 months; childhood, lasting an additional 2 to 4 years; a juvenility stage of 3 to 4 years of semi-independence, followed by adolescence, which lasts 3 to 5 years; and a youth stage, which lasts an average of 4 years [2].

The uniquely human adolescent growth spurt is often regarded by physical anthropologists and ape biologists as the operational definition of adolescence [7], even though it starts before the emergence of secondary sexual characteristics in girls, and much later than the onset of genital changes in boys. There is no evidence for such a human-like adolescent growth spurt in any living ape. With obvious limitations as to what can be inferred from fossilized skeletal remains, there is some suggestive evidence that 1,800,000 years ago hominids may have had a pattern of growth indicative of an adolescent stage of development [8].

In addition to being a period of rapid growth, adolescence is a time of subcutaneous fat deposition, especially in girls. Whereas subcutaneous fat is evenly spread over the female chimpanzee's body, the human adolescent has striking fat deposits in the thighs, buttocks and breasts, even if she is thin overall. These enable her to get through periods of scarcity, signal sexual maturity and facilitate sexual attraction of a mate, and allows others to continuously monitor her nutritional state [9].

### Boys and girls coming of age

Boys and girls embark on different come-of-age strategies to achieve their fertility goals (Table 2). The onset of puberty in girls is generally considered to take place when breast buds erupt (thelarche), but even during childhood and juvenility girls have active ovaries that generate estrogens [10]. It is now recognized that thelarche is not the first sign of maturation of the female hypothalamic-pituitary-gonadal axis. Very much like boys, whose gonads are assessed via direct palpation and show testicular growth before sex-steroid concentrations increase, the ovaries start to grow discretely about two years before thelarche; estradiol levels increase during this period also [11]. Growth acceleration in the girl occurs some 6 months before the budding of breasts, and menarche about a year after the peak height velocity.

**Table 2:** Adolescence in boys and girls manifests differently with regard to their actual fertility.

Boys	Girls
Become fertile approximately 2 years after onset of growth spurt; 1 year before peak height velocity	Completed half their breast and pubic hair development by peak height velocity; menarche approximately 1 year after peak height velocity
Remain juvenile in body hair, stature, muscularity and voice	Appear feminine, while remain infertile
Muscle spurt and adult stature still four years away; approximately 18 years	Adult frequency of ovulation and adult size of birth canal; approximately age 18 years
Learn adult social roles while sexually mature, but not perceived mature by adults	Learn adult social roles while infertile, but perceived by adults as mature

From this time forward, girls have an apparent womanly body form, yet are not fertile; they will develop an adult cycle of ovulation and adult size of the birth canal much later, around 18 years of age. They gain knowledge of their adult social roles while still infertile, but perceived by adults as mature. The perception of fertility in girls facilitates their entry into the social-economic-sexual world of adult women, allowing them to practice reproductive skills without conceiving [12].

Boys show a pattern of gradual maturation of the hypothalamic-pituitary-gonadal axis similar to that of girls, becoming fertile at 14 to 15 years on average, about two years after their peak height velocity. But they are still young in outward development, body size, voice and facial features. Boys will learn their adult social-economic-sexual roles while already sexually mature but not yet perceived as such by adults. This allows them to interact and learn from older adolescents and adults without seeming to compete for status and other important resources, including fertile females [7,13]. Testosterone, which plays a central role in male peak growth velocity, appears to be important for activation of the courtship behavior that leads to the formation of sexual pairing bonds [14].

## Before and during adolescence

### Preceding adolescence - the juvenile stage

**Figure 1:**
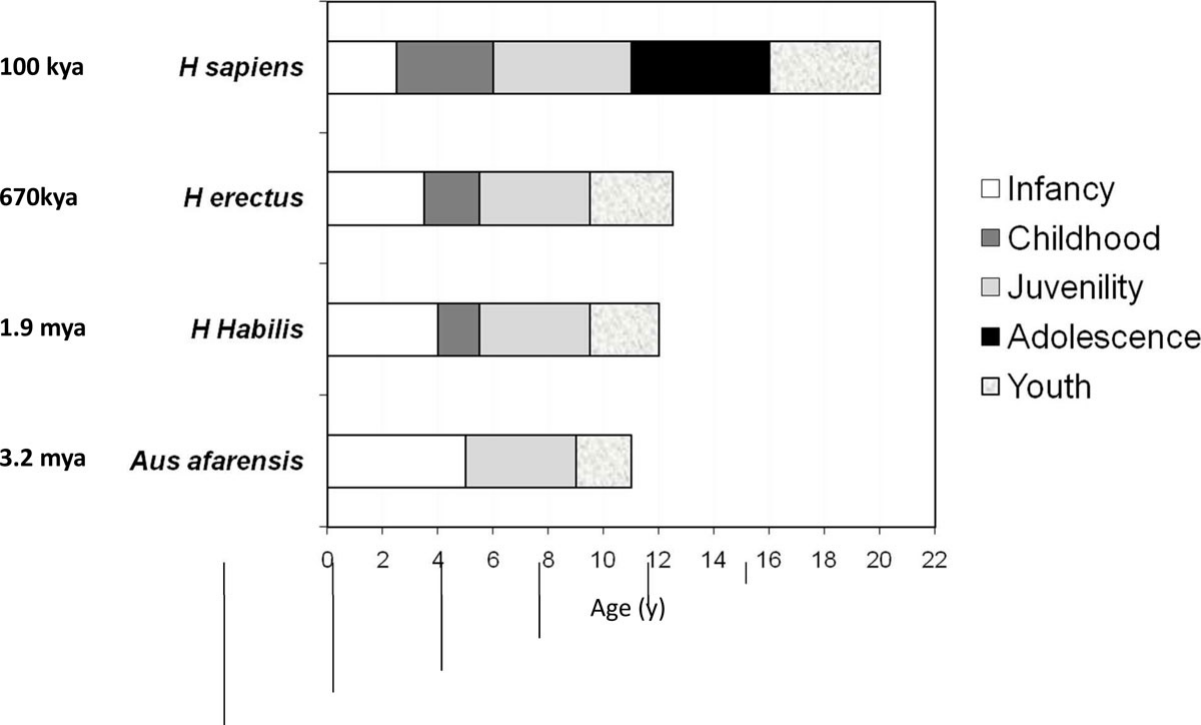
**Evolution of the hominidae life history during the first 20 years of life**. During evolution, childhood and adolescence have been added as new life-history stages and compared with apes and the presumably early hominidae. The chimpanzee serves as a living representative of the assumed *Australopithecus afarensis *life history. As childhood emerged and prolonged, infancy has gradually become shorter, and the latest introduced adolescence came at the expense of a shorter juvenility. Detailed accounts of these stages are given in [2].

All mammals, including the great apes, transit directly from infancy to juvenility without passing through the childhood stage - except humans (Figure 1). Comparison with African apes suggests that the timing of the transition to juvenility, as measured by adrenarche, may be similar to that in humans, although the full course of age-related changes in dehydroepiandrosterone sulfate (DHEAS) and their relationship to reproductive and brain maturation are not clear [15].

We and others have defined juvenility as a distinct life-history stage in humans, characterizing it in terms of endocrine and body composition changes resulting in changes to social assignments and psychological maturation [16,17]. Developmental psychologists refer to this period as 'middle childhood', 'the five-to-seven-year shift' and 'the age of reason and responsibility' [18]: the brain reaches its final size - even if neuronal development is not complete - and primates, equipped with adult molars, forage independently for food and care for themselves. In modern societies the transition to juvenility coincides with the age when children go to school and compete to some extent with adults for food and space, while working out their social standing among age-mates. Coinciding with participation in adult social activities, juveniles develop a strong odor during the juvenile period; intriguingly, olfactory aversion emerges in the case of father-daughter and brother-sister, but not other family relationships, presumably for incest avoidance [19].

Del Giudice contends that juvenility (adrenarche) represents a 'switch point', a time when the environment can reprogram nascent reproductive strategies established earlier in life [17,20]. Indeed, he argues that sex differences in attachment relationships emerge in middle childhood and have adaptive significance for sexually selected life-history strategies. Early psychosocial stress and insecure attachment during juvenility direct development towards mating-oriented reproductive strategies; insecure males tend to adopt avoidant reproductive strategy, whereas insecure females tend to adopt an anxious/ambivalent strategy (which maximize investment from kin and mates). Strategies such as those involving bearing few or many offspring are passed to future generations [17].

In social terms, juvenility offers opportunities to prepare for the social complexities of adolescence youth and adulthood - in part by assaying one's social status and standing in the competitive world of peers [17]. Transition from childhood to juvenility is marked by the onset of adrenal androgen generation (adrenarche), adiposity rebound, deceleration of growth [21], and the eruption of the first molar teeth [16]. Whereas humans and chimpanzees exhibit adrenarche, other primates such as the baboon and rhesus monkey do not, and the adrenals of most other mammals produce little or no DHEA [22]. Thus, adrenarche is a recent evolutionary event. The human and chimp DHEA-generating enzymes, 17,20-lyase, differ at only two amino acids, whereas the human/chimp enzyme differs from the baboon or rhesus enzyme by 25 to 27 residues (95% identity) [22]. Serum DHEA and DHEAS rise progressively throughout juvenility [23], with effects on a wide variety of physiological systems, including neurological [24]and immune [25], as well as somatic growth and development [21,26]. DHEA in humans operates as a neurosteroid, affecting neurological functions and modulating mood [27,28].

The age at transition from childhood to juvenility has been remarkably constant, especially when compared to the plasticity that characterizes other life-history features such as age of sexual maturation [29]. Comparison with the African apes suggests that the timing of adrenarche and the sex difference in chimpanzees timing of transition from infancy to juvenility may be similar to that of humans moving from childhood to juvenility [15,30]. Assuming an important role for adrenarche in human brain maturation, Campbell argued that the increased brain size and extended lifespan of humans relative to the great apes imply changes in the timing and impact of adrenarche [15]. Thus, increases in body size evident among *Homo erectus *imply increases in lifespan and delayed adrenarche and reproductive maturation, and as such are a natural point at which to consider the potential role of delayed transition from childhood to adrenarche in human evolution.

### Transition from juvenility to adolescence

Age and size at adolescence have strong effects on an individual's fitness because they affect reproductive potential, schedule and efficiency [31]. Emphasizing fitness goals, early transition to adolescence and the abbreviation of its duration increase the likelihood of reproduction before death, so may prove especially adaptive under conditions of ecological risk (provided nutrients are sufficient to foster maturation). Accelerated pubertal development also reduces generation time, while potentially lengthening reproductive lifespan. Alternatively, late transition into adolescence lengthens preadolescent growth and the opportunity to embody or internalize the various resources to which the individual is exposed, be those resources nutritional, social or psychological. At the same time, delayed maturation prolongs the preadolescent hazard period, which may be compensated for by continuing parental care. Ultimately, individuals face a tradeoff between maturing to reproduction young and small and maturing at large body size, since for any given growth rate earlier maturation implies smaller size at transition.

## Adaptive developmental plasticity

### The changing age of adolescence

Substantial physiological variation of some 4 to 5 years is evident for age at onset of sexual maturation across individuals under varying life conditions [32]. Despite substantial heritability in pubertal development [33,34], much variation remains to be explained [34], leaving room for the influence of environmental factors to adjust the phenotype in the service of fitness goals [32].

**Figure 2:**
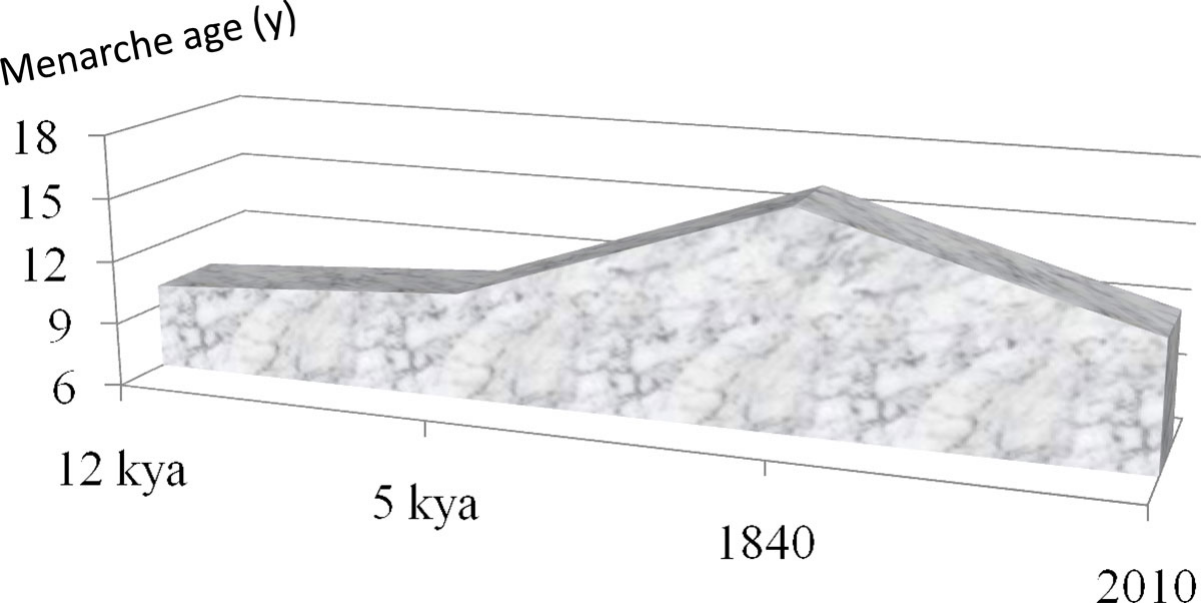
**Menarche age over the last 12,000 years**. The age of menarche gradually increased until the recent secular trend's decline, as shown in Figure 3. Data from [36].

The human population has grown exponentially from a brink of extinction 80,000 years ago, with a world population of several thousands, to that of several billion today. Such population growth increased opportunity for genetic mutations, thereby accelerating the pace of human evolution [35]. Estimates indicate that the age for menarche around 20,000 to 12,000 years ago - at the beginning of the agrarian period - was 7 to 13 years, and that full reproductive competence in Neolithic females (New Stone Age, 12,000 to 5,000 years ago) occurred at age 9 to 14 years (Figure 2) [36]. This would place menarche at 7 to 12 years, assuming a 2- to 4-year gap between menarche and reproductive competence [37]. This suggests that menarche in Neolithic times occurred even earlier than observed in modern Western countries [36]. This claim is consistent with data on the Aeta of the Philippines, who reproduce as early as age 10 to 14 years [38].

**Figure 3:**
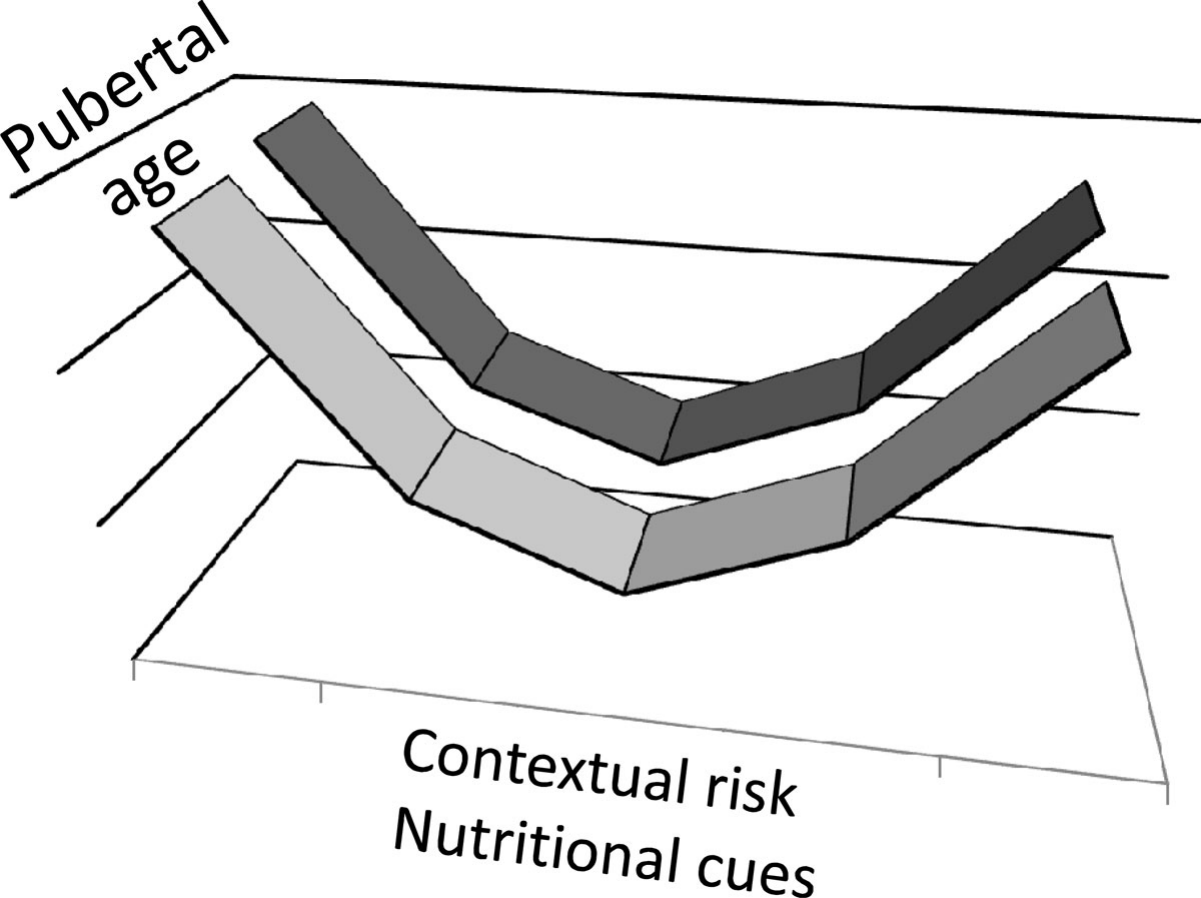
**The U-shaped link between contextual risk and nutritional cues as they predict pubertal development**. When immature animals experience severe environmental stresses such as malnutrition or disease, maturation is often delayed until conditions improve and normal growth can resume. By contrast, when animals are raised under ideal conditions that promote rapid growth, internal checkpoints ensure that maturation does not occur until juvenile development is complete. But when contextual stress is not so great as to challenge survival, pubertal development is accelerated, thereby increasing the likelihood of reproduction before death or disability.

Developmental and maturational tempo is flexible and responsive to environmental conditions in a presumptively adaptive manner. When immature animals experience severe environmental stresses such as malnutrition or disease, maturation is often delayed until conditions improve and normal growth can resume. By contrast, when animals are raised under ideal conditions that promote rapid growth, internal checkpoints ensure that maturation does not occur until juvenile development is complete. But when contextual stress is not so great as to challenge survival, pubertal development is accelerated, thereby increasing the likelihood of reproduction before death or disability. Collectively, these phenomena highlight a U-shaped link between contextual risk and nutritional cues as they predict pubertal development (Figure 3). Nutritional cues have clear temporal influence, including the timing of juvenility and adolescence, with a trend towards earlier maturation among those whose average body mass early in life is lower or higher than average, yet later among those with a poor childhood weight gain [32], resulting in a U-shape relationship [39,40].

Domestication of animals and agriculture altered the human environment - and, thereby, human development - in several ways, including adaptive changes in the onset of puberty. A relatively sedentary lifestyle increased local human densities, facilitated the spread of infectious diseases and was associated with recurrent famines. Later maturation to adulthood was a tradeoff to adapt to poor nutrition as well as the increasing complexity of being an adult in a society engaged in agriculture, settlement and population aggregation. Increased differentiation of social tasks and the creation of societal hierarchies in wealth-accumulating agrarian societies resulted in variation in nutritional status and family conditions, which themselves led to an overall increase in the mean age of menarche. This crucial point is further discussed below under 'Evolutionary Theory of Socialization'. Thus, by medieval times, the average age of menarche was deferred to 16.5 years, as it remains today among underprivileged adolescents in developing countries [32].

Modern hygiene and medicine have influenced nutritional and infectious constraints on puberty, resulting in the secular trend in pubertal development over the past 150 years (see below). Further evidence to this effect would seem to come from research showing that girls and, to a lesser extent, boys adopted from developing to industrial countries show accelerated sexual development [41,42]. The greater tendency of adopted girls to respond with pubertal onset to a changing environment is in line with female preponderance of idiopathic central precocious puberty. As note earlier, it is the female who has intrinsic constraints on the number of offspring she can generate over her reproductive years, and it is females more than males who may enjoy a fitness advantage from early maturation. Thus, evolutionary life-history thinking challenges the notion that earlier puberty is the result of a hypothalamic control malfunction, viewing it rather as an adaptive response to improving life conditions, similar to that witnessed in the case of the secular trend.

The Philippine Aeta provide unique insight into the strategic importance of the age of adolescence. Their growth deviates from the U.S. 0.01^th ^percentile, showing an early juvenile deceleration, early pubertal spurt and early growth termination [38]. With life expectancy of 16 and 27 years at birth and adulthood, respectively, their first reproduction occurs at age 10. Early reproduction minimizes the likelihood of death before reproduction. Thus it seems that early fecundity evolved to adapt to high-risk, high-mortality life of a short duration, with shortness of stature resulting from a short period of preadolescent growth [38].

### The secular trend in pubertal maturation

**Figure 4:**
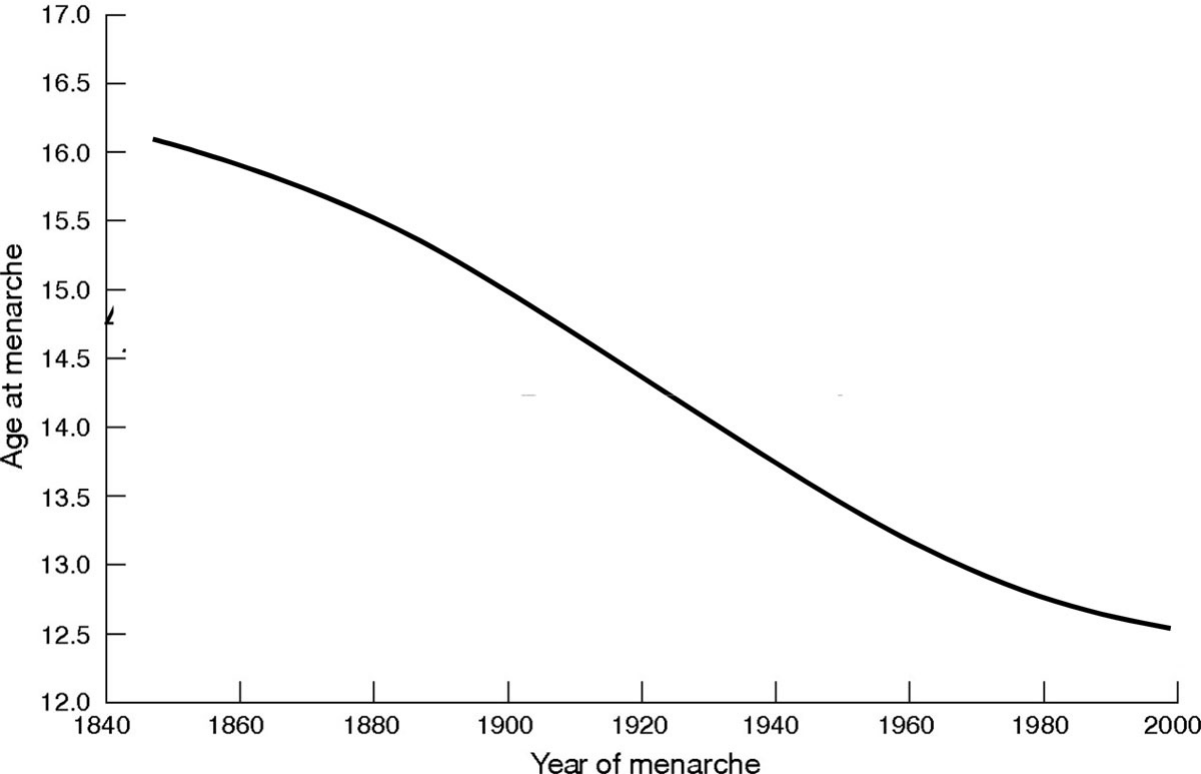
**The secular trend in puberty**. Declining age of menarche in Western societies from 1840 to 2000. Data from [68]. The line does not show a saturation point; the trend is expected to continue.

The secular trend provides compelling evidence that pubertal development is developmentally plastic. Over the past 150 years - without any documented change in gene frequencies - the age of menarche has fallen by a full 4 years in the industrialized West (Figure 4).

As much as the secular trend in human size has been an adaptive response to a nutritionally rich environment, the receding age of adolescence and pubertal development has been an adaptive response to positive environmental cues in terms of energy balance. The ever-younger age of girls' thelarche and menarche may have more than a single justification, however. In the last decade, the popular explanation has been that this phenomenon results from environmental endocrine disruptors that accelerate hypothalamic maturation [43]. Whereas endocrine disruptors may have a bearing on the earlier age of thelarche, which is a recent trend, it can hardly explain the secular trend in the age of menarche over the last 150 years.

Following Belsky *et al. *[20] and Gluckman and Hanson [36], we explicitly challenge the concept that this has been a disease process, proposing that accelerated pubertal development reflects contextually regulated reproductive and life-history strategies. Indeed, the age at transition from juvenility to adolescence in humans has a variety of physical and social correlates. Women face a tradeoff between spending a long time accumulating resources through childhood growth, thereby improving the odds for successful pregnancy while also risking death before sexual maturation, against beginning early reproduction and increasing the number of reproductive cycles. A later first birth allows for a longer period of adolescent weight gain, and heavier women in traditional societies are more fertile; both these attributes correlate with higher birth rates. This tradeoff has been used to model the optimal age at first birth, which under such conditions is 18 years, near the observed mean of 17.5 years in such societies [44].

### Evolutionary theory of socialization

**Figure 5:**
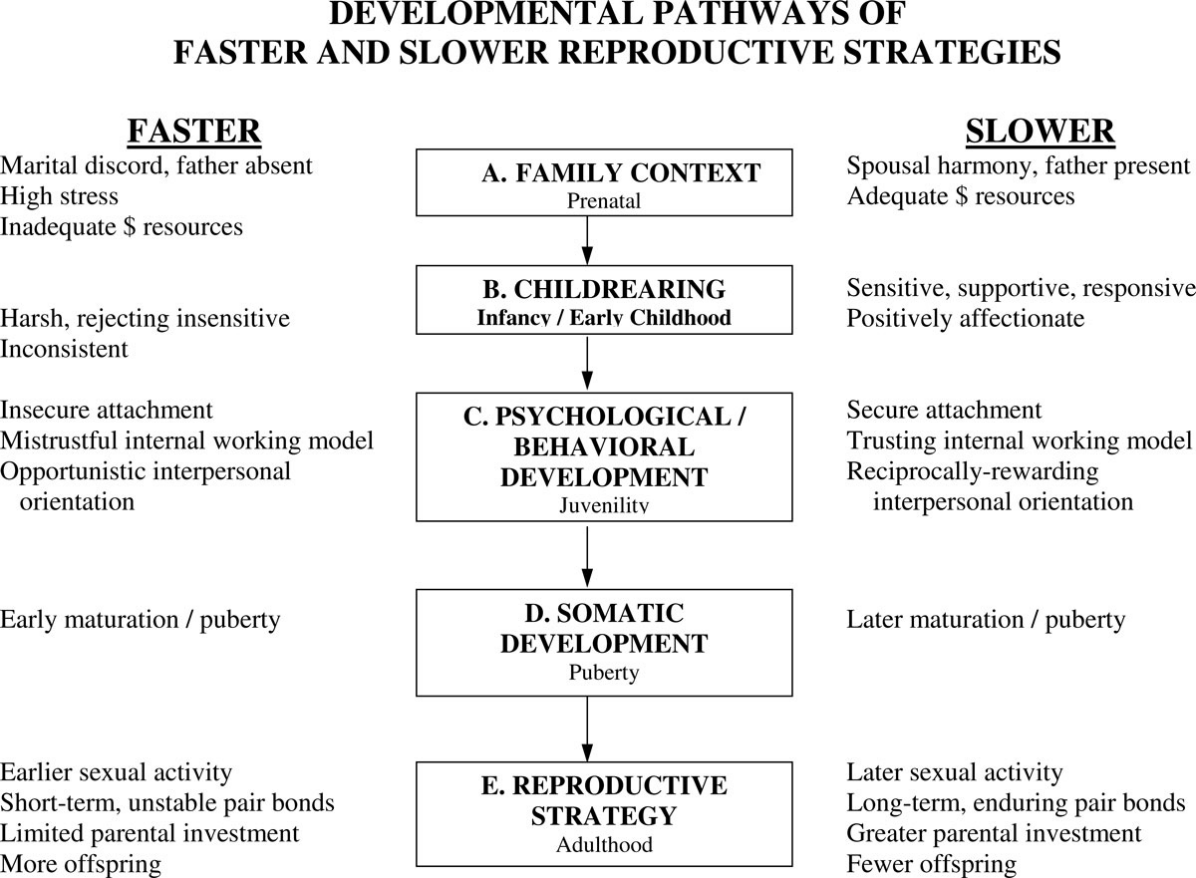
**Faster and slower reproductive strategies: Reproductive strategies develop in different contexts and are characterized by diverging patterns of psychological, somatic and behavioral development**. More and less supportive family contexts (and broader ecologies) influence the quality and quantity of parental investment, which in turn influences psychological and behavioral development. Collectively these forces regulate the timing of pubertal development and, thereby, sexual behavior, pair bonding and eventual childbearing and parental investment. The faster strategy fits a world in which risk and uncertainty is high, whereas the slower strategy fits a world in which resources are predictably available and sufficient. The faster strategy enables the individual to reduce the risk of dying before reproducing and reflects the fact that the individual's capacity to attract and maintain a high-quality mate and provide resources to their own (eventual) offspring will be limited. The slower strategy reflects the opposite. Based on [20].

Belsky *et al. *[20] advanced an evolutionary theory of socialization stipulating that familial psychosocial stress (for example, marital conflict, harsh parenting, father absence), itself induced by extrafamilial ecological stress (for example, limited income, unemployment), fosters a fast life-history and reproductive strategy. They claimed that pubertal maturation played a previously unappreciated role in linking early rearing experiences with subsequent mating and parenting, in line with the attachment theory of Bowlby [45,46], which is hereby expanded (Figure 5). The evolutionary reasoning was that early maturation would be selected under conditions of emotional risk and uncertainty, thereby setting the stage for earlier sexual debut, more promiscuous mating and the bearing of more offspring, along with lesser parental investment. Natural selection favors accelerated development when early life experiences suggest an insecure world in which intimate relations are not enduring [47]. Thus, slower physical maturation would risk lowering reproductive fitness and survival: in an insecure world, maturing early and breeding promiscuously would enhance reproductive fitness more than delaying development, mating cautiously and investing heavily in parenting. The latter would make evolutionary sense for reproductive fitness in a secure world, as perceived by the young child and juvenile [48]. Such theorizing is certainly consistent with evidence that earlier pubertal development is associated with greater sexual risk taking; earlier age of menarche is associated with earlier age of first dating, first kissing, first genital petting and first sexual intercourse, and higher rates of adolescent pregnancy, as reviewed [49,50].

In the two or more decades since the adaptive-predictive-response theory of human development by Belsky *et al. *appeared, an abundance of evidence consistent with its critical pubertal-timing prediction has emerged (for review see [51]). Consider in this regard findings from longitudinal research indicating that limited family support during childhood (for instance, authoritarian parenting, negative family relationships) is associated with females' advanced adrenarche and early puberty [52], and that harsh parenting in early childhood predicts earlier age of menarche and, thereby, greater sexual 'risk taking' in adolescence [53]. Important also are data showing that younger sisters with greater earlier exposure to an absent father as a result of divorce or separation matured earlier than did their older sisters [54], and that girls evacuated from their homeland during World War II and sent to live in Sweden and Denmark reached menarche at a younger age and even bore more children than members of the same birth cohort who remained at home [55].

In line with the above, experimental manipulation of licking and grooming of newborn rats by their mothers illuminates the role of epigenetic processes in regulating the stress-response system, pubertal timing, sexual behavior and parenting [56], as reviewed [51].

### Individual differences in developmental plasticity

Some individuals are more plastic and responsive to environmental cues and others less so, adopting a more fixed developmental trajectory for reproductive strategy [50,57-62]. Children who were more physiologically reactive in terms of cortisol response to a psychological challenge were more responsive to family forces in accelerating pubertal development [63]. In a recent gene-environment interaction study, an allelic variation in the estrogen-receptor gene determined which girls' menarche age was accelerated by high levels of family conflict [64]. Such findings suggest that population estimates of environmental influences on pubertal development do not necessarily reflect individual response.

## Conclusions

This review uses an evo-devo approach and life-history theory for understanding human adolescence and especially variation in the timing of reproductive maturation. Developmental and maturational traits that respond to environmental cues enhance fecundity-survival schedules and behavioral strategies that yield the highest fitness in a given environment.

Why is it that we have a unique adolescence phase, preceded by a juvenile phase and followed by a youth phase leading to such a delayed reproduction? Like other organisms, humans evolved to withstand environmental hardships by responding in ways that maintain evolutionary fitness, even if submaximal. The means to do this is a series of predictive adaptive responses that utilize the sensitive times of transitions from one life-history stage to the next, each assigned with its own domain. The transition from juvenility to adolescence is deferred when food supply is short, programming at the same time for later fecundity and fertility and for longevity [65,66].

The timing of puberty sheds light on the relationship between phenotypic adaptive plasticity and adaptive genetic changes. Whereas the gradual tendency to mature late over *Homo*'s evolution is genetic, the gradual secular trend in industrial societies over the last 150 years is not, given the short timescale. The more recent tendency for earlier puberty reflects the overall quality of modern environments, allowing females to approach the extremes of their genetic range of reaction. Such evo-devo thinking demands reconsideration of the notion of 'precocious puberty'. This term implies pathology, whereas the vast majority of early puberty probably reflects normal physiological and adaptive developmental plasticity [36]. Thus, the term precocious puberty is to be reserved for those few with anatomical or genetic defects, without a precise definition in the present context.

The implications of disconnection of the mental and somatic components of human adolescence are underappreciated, and result in both mental and somatic consequences. Among them are the obesity and polycystic ovary syndrome epidemics, but also mental and social behaviors. In an American study, early-maturing girls displayed higher levels of self-reported criminality, drug abuse, social isolation, early sexual behavior and psychiatric problems [67]. Early-maturing girls, particularly those with a history of adolescent conduct disorder, were more likely to be depressed and to have many sexual partners in young adulthood compared with their counterparts. Early puberty may thus represent a social pathology in industrialized societies. The claim we make, however, is markedly different from the widespread assertion that early maturation is a risk factor for aggression and delinquency [68]. Rather, we contend here, that early life experience during infancy and childhood will be associated with a change in maturational tempo, such that harsh rearing conditions predict earlier age maturation and associated behavioral phenotypes, including perhaps aggression, delinquency and promiscuity because these responses were selected to promote fitness. This framework is quite distinct from the disease perspective that fails to appreciate the evolutionary wisdom of maturing early and behaving in opportunistic, advantage-taking ways under certain contextual conditions.

Hereditary, environmental and stochastic factors regulate puberty in a unique environment, but their relative contribution to the phenotypic outcome and the extent of stochastic epigenetic reprogramming that is required to alter human phenotypes is not known because few data are available [69]. If the environment can influence developmental and maturational trajectories during pre-adult life-history stages, how do epigenetic events influence the transition from one life-history stage to the next, growth and puberty at the molecular level? Growth and puberty are regulated by insulin, growth hormone, the insulin-like growth factors and the sex hormones, to mention a few of the control factors. These hormones drive the rate of growth and development, but it is unclear how the environment shapes the timing of the different phases of developmental events. Epigenetic mechanisms potentially play an important role.

Perhaps the most fundamental question raised by the life-history approach to adolescence concerns the uniqueness of each child in her given genetic background and current environment as they best serve her reproductive fitness. Given the evidence on the strong influence of socioeconomic conditions early in life, we have to better understand how these interact with or via endocrine mechanisms to generate signals that affect life history and adolescence.

## Abbreviations

DHEA: dehydroepiandrosterone; DHEAS: dehydroepiandrosterone-sulfate.

## Competing interests

The authors declare that they have no competing interests.

## Authors' contributions

Both authors conceived of the article and wrote it. Both authors read and approved the final manuscript.

## Pre-publication history

The pre-publication history for this paper can be accessed here:

http://www.biomedcentral.com/1741-7015/11/113/prepub
